# A Proposition:—Relating to the Modification and Prevention of Certain Zymotic Diseases

**Published:** 1869-02

**Authors:** Theodore Griffin

**Affiliations:** 789 State Street, Chicago


ARTICLE VIII.

### A PROPOSITION:—RELATING TO THE MODIFICATION AND PREVENTION OF CERTAIN ZYMOTIC DISEASES.

BY THEODORE GRIFFIN, M.D., 789 State Street, Chicago.

*Those diseases which, after once being developed in the human system, thereby destroy the susceptibility of the system to a second like invasion, may be prevented, or their severity greatly modified, by the introduction into the human system of a like virus, transferred from the lower orders of the animal creation.*

The diseases which may thus be modified or prevented, belong to the class known as "Zymotic;" and are small-pox, scarlatina, measles, etc.; including the whole catalogue of diseases alluded to in my proposition.

The modification and prevention of—once the greatest scourge of mankind—small-pox, by the introduction into the human system of a similar virus, taken usually from the cow, leads fairly to the inference set forth in the foregoing proposition. It has been observed that the cat, horse, and many of the lower animals, are subject to eruptive and other diseases; but I am not aware that careful inquiry has been made into the pathology of these morbid conditions, which, it may be found, have their prototype in the human system. The disease observed in cows, called cow-pox, is identical with small-pox, and affects alike cows, horses, and monkeys; and human small-pox may be communicated to these animals by the impalpable emanations (the specific effluvia) through the medium of the atmosphere.

May we not discover other of the diseases alluded to, among the lower animals? And if so, may we not hope to obtain results similar to those which follow the introduction of the cow-pox virus into the human system—namely, *exemption from these diseases?* The subject deserves investigation. If we are not able to detect, for example, scarlatina in the cat or dog, we may readily test his susceptibility to the disease by inoculation with the specific poison. Should disease develop itself as a result, we may fairly conclude that we have scarlatina: now we may transfer the virus to the human system, and await the result; which result we may expect to be a modified form of the disease, with complete exemption from a future attack.

Let us thus open an avenue through which we may thrust those scourges of our race out of the world, as the immortal Jenner virtually thrust out small-pox, but failed to see the general application of the principle upon which it was accomplished.

